# Micropipes in SiC Single Crystal Observed by Molten KOH Etching

**DOI:** 10.3390/ma14195890

**Published:** 2021-10-08

**Authors:** Hejing Wang, Jinying Yu, Guojie Hu, Yan Peng, Xuejian Xie, Xiaobo Hu, Xiufang Chen, Xiangang Xu

**Affiliations:** 1 State Key Laboratory of Crystal Materials, Shandong University, Jinan 250100, China; hejingwang@mail.sdu.edu.cn (H.W.); yujinying@mail.sdu.edu.cn (J.Y.); 2 Institute of Novel Semiconductors, Shandong University, Jinan 250100, China; guojie_hu@mail.sdu.edu.cn (G.H.); pengyan@sdu.edu.cn (Y.P.); xbhu@sdu.edu.cn (X.H.); xxu@sdu.edu.cn (X.X.)

**Keywords:** SiC, micropipes, KOH etching, classification of etch pits, Raman

## Abstract

Micropipe, a “killer” defect in SiC crystals, severely hampers the outstanding performance of SiC-based devices. In this paper, the etching behavior of micropipes in 4H-SiC and 6H-SiC wafers was studied using the molten KOH etching method. The spectra of 4H-SiC and 6H-SiC crystals containing micropipes were examined using Raman scattering. A new Raman peak accompanying micropipes located near −784 cm^−1^ was observed, which may have been induced by polymorphic transformation during the etching process in the area of micropipe etch pits. This feature may provide a new way to distinguish micropipes from other defects. In addition, the preferable etching conditions for distinguishing micropipes from threading screw dislocations (TSDs) was determined using laser confocal microscopy, scanning electron microscopy (SEM) and optical microscopy. Meanwhile, the micropipe etching pits were classified into two types based on their morphology and formation mechanism.

## 1. Introduction

Silicon carbide (SiC), a typical representative of the third-generation semiconductor materials, has unique properties such as wide band gap, high breakdown voltage, high thermal conductivity and excellent chemical inertness [1,2,3,4]. These characteristics are different from traditional semiconductors such as silicon (Si) and gallium arsenide (GaAs), making SiC suitable for fabricating high-power and microwave radio-frequency devices [5]. In recent years, the technology for preparing SiC substrates has become increasingly mature. Some breakthroughs have been made, especially in suppressing micropipes generation [6]. As a super-screw dislocation, it has always been difficult to accurately distinguish micropipes from threading screw dislocations (TSDs). According to Frank’s theory, micropipes are interpreted as hollow-core tubes extending along the c-axis, and are clearly detrimental to high-power devices [7,8]. Therefore, the characterization and analysis of micropipe defects in SiC single-crystal material has become an important issue for the improvement of the single-crystal growth process and the preparation of high-performance devices. Tomohisa Kato et al. determined the photoelastic constant in the plane of (00$\bar{1}$) 6H-SiC and then estimated the magnitude of the internal stress around the micropipes [9]. A unique transitional configuration of superscrew dislocations to closed-core elementary screw dislocations was proposed by Daisuke Nakamura et al., which showed good ability to explain the decrease of micropipe density during crystal growth [10]. Arora, Aman, et al. used cathodoluminescence (CL) imaging technology to confirm that multiple micropipes could originate from a single hexagonal void, and energy-dispersive spectroscopy (EDS) showed that the inside of the micropipe walls exhibited higher levels of carbon [11]. However, some research questions on micropipes have yet to be successfully examined. In particular, accurately distinguishing micropipe etch pits from TSDs after wet etching has also been a technical problem. In addition, Raman spectroscopy is becoming an increasingly common analysis method, and offers fast and contact-free measurements with easy sample preparation. Raman spectroscopy is a vibrational spectroscopy method based on the analysis of inelastically scattered light. It has been applied to SiC materials for the characterization of polytypes [12], stacking faults [13], stress [14] and doping [15]. Shenghuang Lin et al. used Raman scattering to study the spectra on the Si surface of 6H-SiC crystals including micropipes, and the second-order Raman features of the micropipes in bulk 6H-SiC were well-defined using the selection rules for second-order scattering in wurtzite structure [16]. Following these, few papers have been published focusing on this topic. Therefore, it is necessary to apply Raman spectroscopy to the characterization of micropipe etch pits. In the present work, some new discoveries appear in the Raman spectra of micropipe etch pits.

Selective chemical etching has been extensively used to study defects in SiC single crystals, with methods such as wet etching [17,18,19,20,21], hydrogen etching [22,23,24], dry etching [25,26,27] etc. In these approaches, KOH and its mixtures with other salts are currently preferred for revealing defects and dislocations in SiC single crystals. Robert T. Bondokov et al. used KOH steam to etch the (0001), (000$\bar{1}$), (11$\bar{2}$0) and (1$\bar{1}$00) surfaces of SiC [28]. However, this method needs to maintain the temperature at 700–1000 °C under normal pressure. This high temperature limits the application of this method. Yong-Zhao Yao et al. used KOH and Na_2_O_2_ as an etchant (KN etching) to identify the types of dislocations in SiC [29]. This method is only suitable for n-type SiC substrates with an off-angle of 0° to 8°. Sandeep Mahajan et al. utilized molten KOH to etch 6H n-type SiC single-crystal wafers [30]. Their results revealed that 500 °C was the optimum temperature for the identification of micropipes (MPs), threading screw dislocations (TSDs), threading edge dislocations (TEDs) and basal plane dislocations (BPDs). Despite being a destructive technique, the molten KOH etching method is still considered an effective method to characterize dislocations in SiC [31,32,33,34,35,36,37,38]. 

In this work, we take advantage of the method of defect-preferred corrosion and molten KOH, successfully determining the etching condition to distinguish micropipe etch pits from TSD by etching 4H-SiC and 6H-SiC wafers for different amounts of time. The results demonstrate that under-corrosion is a suitable condition to form micropipe etch pits apart from TSDs. We classified the micropipe etch pits based on their formation mechanism and morphology. Moreover, the Raman performance of the micropipes are also characterized, and we find a new Raman peak corresponding to micropipe etching pits.

## 2. Experiment

Nominally undoped 4H-SiC and 6H-SiC single crystals were grown on on-axis seeds by the physical vapor transport (PVT) method. After crystal growth, the 4H-SiC and 6H-SiC ingots were processed into standard substrates. The wafers were mechanically polished on both sides. A nickel crucible (inert for molten KOH) was used for KOH heating, and the temperature was set to 460 °C. Diced 4H-SiC and 6H-SiC samples were immersed in molten KOH and etched for different amounts of time. After etching, the wafers were taken out and cooled to room temperature naturally, then washed successively with deionized water and absolute ethanol. A LEXT OLS4000 laser confocal microscope from Olympus (Tokyo, Japan) and a S-4800 scanning electron microscope (SEM) from Hitachi (Tokyo, Japan) were used to observe the morphology of the etch pits on the surface. Meanwhile, the micropipe etch pits were classified based on their morphologies and Raman features of micropipes were examined by a LabRAMHR800 system (Horiba Jobin Yvon, Paris, France) with a 532 nm solid laser as the excitation source. In order to track the changes in Raman spectra, the Raman spectra of micropipes were compared and interpreted before and after different etching times.

## 3. Results and Discussion

### 3.1. The Morphologies of Micropipe Etch Pits and the Etching Rates of Different Polytypes

**Figure 1:**
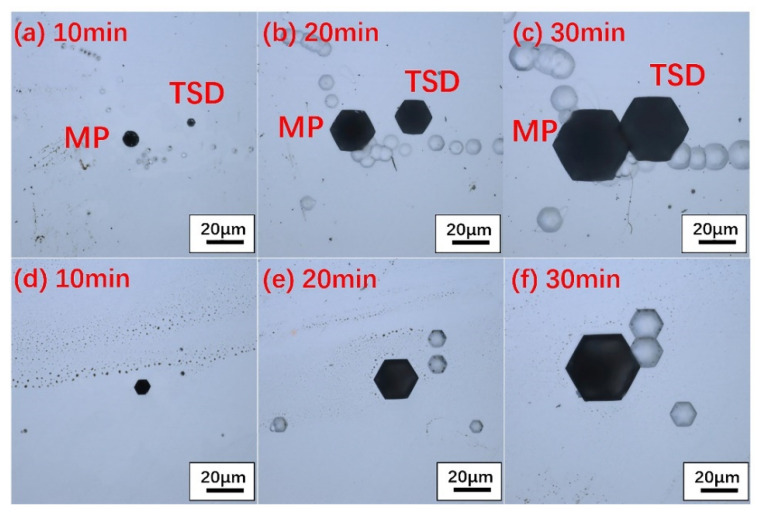
Evolution of the morphology of micropipe etch pits with time in 4H-SiC ((**a**) 10 min, (**b**) 20 min, (**c**) 30 min) and 6H-SiC ((**d**) 10 min, (**e**) 20 min, (**f**) 30 min) under a laser confocal microscope.

**Figure 2:**
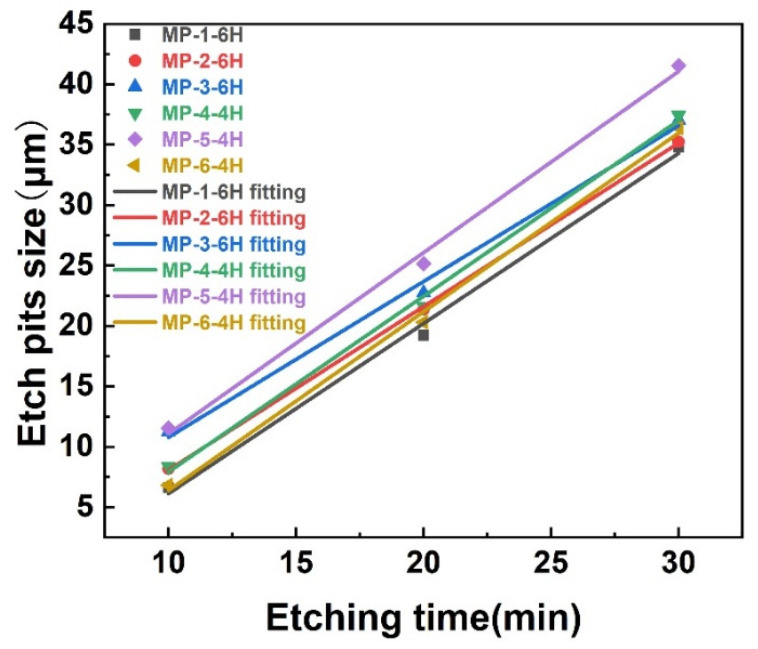
Etch pits size of micropipes vs. etching time.

The 4H-SiC and 6H-SiC wafers were etched at 460 °C for different amounts of time. The specific etching situation is shown in Figure 1. It is obvious that as the etching time increased, the size of the micropipe etch pits also increased. By measuring the size of the etch pits of the micropipes at different etching times, it was found that the size of the micropipe etch pits had a linear relationship with the etching time, as shown in Figure 2. After etching for 10 min, three groups of micropipe etch pits with similar sizes of 4H-SiC and 6H-SiC (MP-1-6H and MP-6-4H, MP-2-6H and MP-4-4H, MP-3-6H and MP-5-4H) were selected. Then, the size changes of the micropipe etch pits of different polytypes were observed through a longer period of etching. The etching rate was measured by the size change of the micropipe etch pits within a fixed time. The polytypic dependence of etching rate was also revealed. There was an increase from the 6H to 4H polytypes in etching rate, which corresponded to their hexagonality, indicating that the etching rate increased as the hexagonality of the SiC crystals increased [39].

### 3.2. Exploration of Etching Conditions to Distinguish TSDs and Micropipe Etch Pits

**Figure 3:**
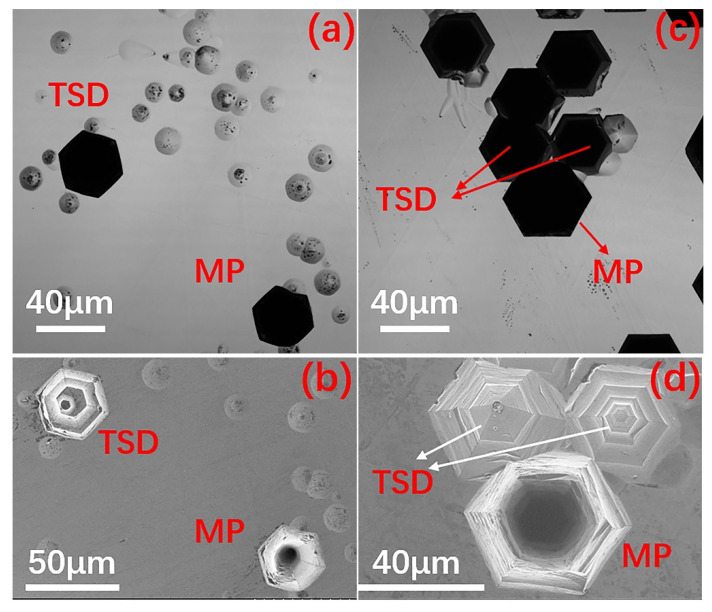
Etch pits of 4H-SiC (**a**,**b**) and 6H-SiC (**c**,**d**) under SEM (**b**,**d**) and laser confocal microscope (**a**,**c**).

The etching pits formed by micropipe defects and TSDs in 4H-SiC and 6H-SiC all appeared as black hexagons under laser confocal microscope, and were difficult to distinguish (see pits marked by red labels in Figure 3a,c). In fact, according to the mechanism proposed by Frank, the micropipes in SiC single crystals are hollow screw dislocations with a large Burgers vector, also known as superscrew dislocations [40]. Since the strain energy associated with the dislocations is proportional to the square of the Burgers vector, the crystal containing the micropipes reduces its strain energy by removing the core of the dislocation [7]. As a result, the center of the micropipe should be a hollow tube. Therefore, the etch pits of the micropipes were bottomless hexagonal etch pits, while the TSDs should be regular hexagonal etch pits with bottoms. This is verified in Figure 3b,d.

**Figure 4:**
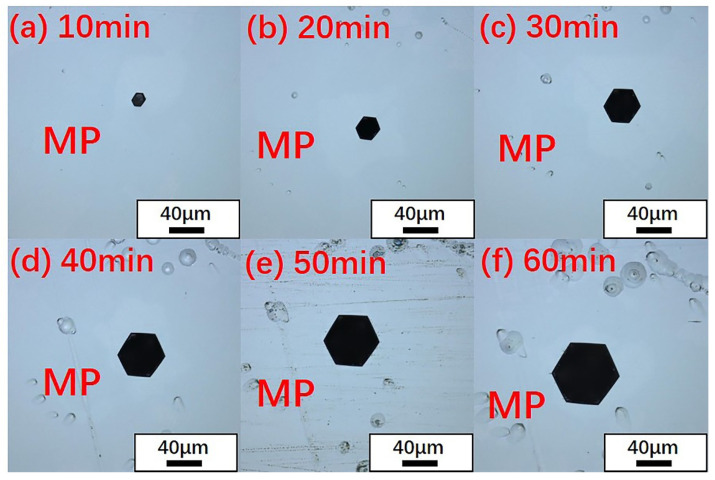
Evolution of the morphology of micropipe etch pits with time under a laser confocal microscope. (**a**) 10 min, (**b**) 20 min, (**c**) 30 min, (**d**) 40 min, (**e**) 50 min, (**f**) 60 min.

**Figure 5:**
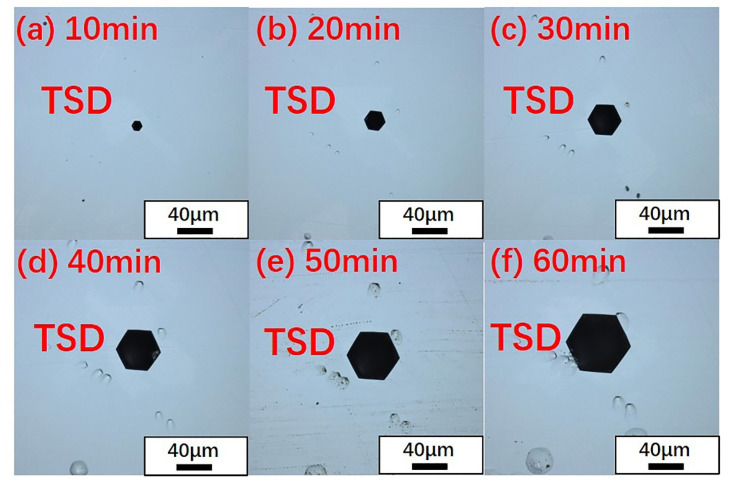
Evolution of the morphology of TSD etch pits with time under a laser confocal microscope. (**a**) 10 min, (**b**) 20 min, (**c**) 30 min, (**d**) 40 min, (**e**) 50 min, (**f**) 60 min.

However, scanning electron microscopy (SEM) was not suitable for the identification of micropipe etch pits in large SiC single crystals. Therefore, careful selection of etching conditions was very important in order to distinguish micropipe etch pits from TSDs under optical microscope and laser confocal microscope. When the wafer was etched at 460 °C for different amounts of time (Figure 4 and Figure 5), it was found that as the etching time increased, the size of the TSDs increased accordingly while the size of the micropipes increased. The difference in size between TSD and micropipe etch pits was also reduced, as shown in Table 1.

**Figure 6:**
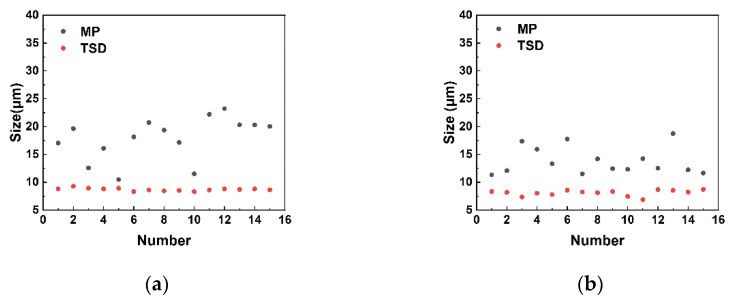
Comparison of micropipe etch pits and TSDs in size: (**a**) 4H-SiC; (**b**) 6H-SiC.

Therefore, 4H-SiC and 6H-SiC were etched for a shorter period. The sizes of micropipe and TSD etch pits were determined. The results showed that the size of TSDs was stably distributed in 7–9 µm, while the size of micropipe etch pits was distributed in a larger range. Notably, the micropipe etch pits were larger than the TSDs, as shown in Figure 6. This was because the micropipe defects have a larger Burgers vector [41]. We believe that the variability of the micropipe etch pits size is closely related to variations in the Burgers vector. A micropipe with a relatively large Burgers vector will produce a larger etch pit. On the contrary, a micropipe with a relatively small Burgers vector will produce a smaller etch pit. The above results can prove that under-corrosion is a suitable condition to identify micropipe etch pits from TSDs.

### 3.3. The Classification of Micropipe Etch Pits

**Figure 7:**
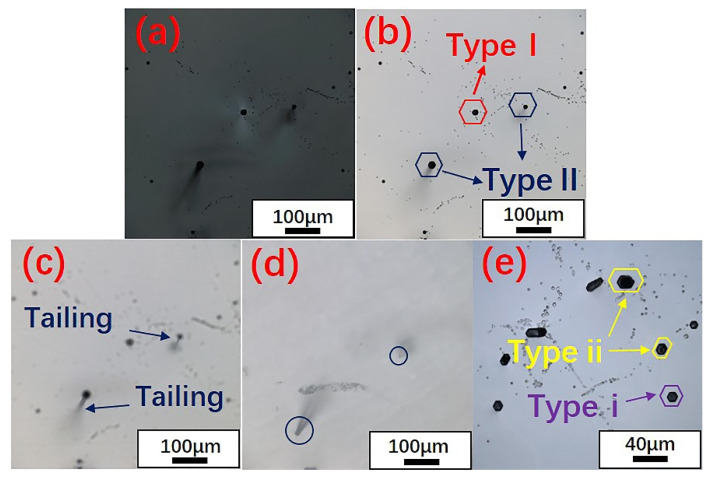
Classification of 4H-SiC micropipe etch pits: (**a**) transmitted polarized microscopic image of the area shown in (**b**); (**b**) Type II and Type I micropipe etch pits observed with a 20× lens; (**c**) tailing of Type II micropipe etch pits when the focal length was changed; (**d**) black spots formed on the back by the Type II micropipe etch pits; (**e**) micropipe etch pits of different shapes observed with a 50× lens.

**Figure 8:**
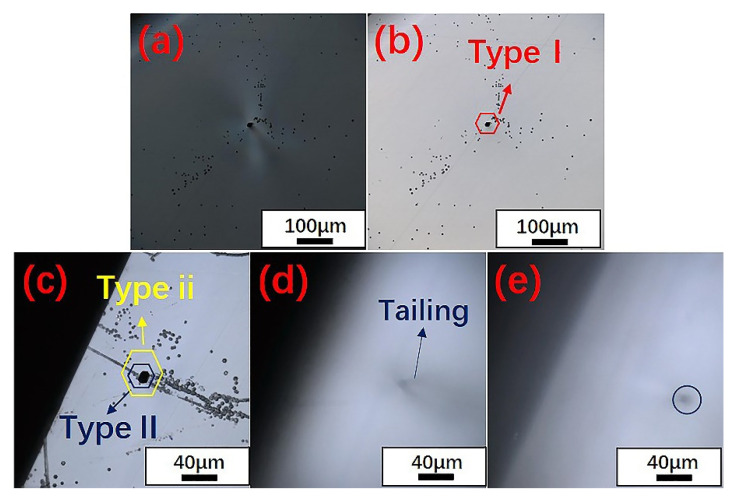
Classification of 6H-SiC micropipe etch pits: (**a**) transmitted polarized microscopic image of the area shown in (**b**); (**b**) Type I micropipe etch pits observed with a 20× lens; (**c**) Type ii and Type II micropipe etch pits observed with a 50× lens; (**d**) tailing of Type II micropipe etch pits when the focal length was changed; (**e**) black spots formed on the back by the Type II micropipe etch pits.

We also found that the hexagonal etch pits formed by the micropipes had different morphologies under the same etching conditions. According to the morphologies of micropipes, two types of micropipe etch pits were observed using laser confocal microscopy, as shown in Figure 7 and Figure 8. In terms of shape, one type (named Type i) was a black regular hexagonal etch pit without a bottom (Figure 7e, Type i), while the other type (Type ii) was a black irregular elongated hexagonal etch pit, as marked in Figure 7e and Figure 8c. It is worth noting that Type i micropipes, regular hexagonal micropipe etch pits, were not observed in 6H-SiC in the under-etched state. Figure 1d–f shows that the shape of the micropipe etch pits changed from irregular hexagon to regular hexagon during the etching process. Because the etching rate of 6H-SiC was lower than that of 4H-SiC, the micropipe etch pits in 6H-SiC remained as irregular hexagons, and did not transform into regular hexagons. Therefore, Type i micropipe etch pits were not found in 6H-SiC. 

According to the formation mechanism of micropipes, areas with high stress form micropipes to relax stress during the growth of SiC single crystals [42]. This led to the unique morphology seen in the transmitted polarized microscopic image of the micropipe, that is, a high-brightness butterfly shape with a black dot in the center [43]. After the etching, the stress around the micropipes had not completely disappeared, and hexagonal etch pits appeared at the micropipe core. Micropipe etch pits were also classified into two types based on this mechanism. Figure 7a and Figure 8a show Type I micropipe etch pits with high-brightness butterfly wings surrounding them, resulting from stress relief. Figure 7b–d and Figure 8c–e exhibit Type II micropipe etch pits without stress relief. Type II micropipes appeared to tail with the adjustment of the focal length of the optical microscope, and would eventually form a black spot on the back of the wafer.

### 3.4. Raman Spectra of Micropipe Etch Pits

**Figure 9:**
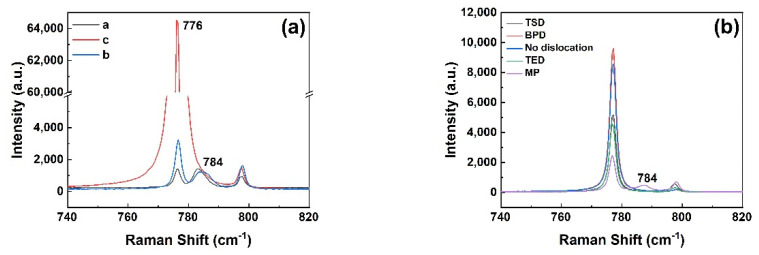
(**a**) Raman spectra of 4H-SiC micropipes; (**b**) Raman spectra of other dislocations and micropipes.

**Figure 10:**
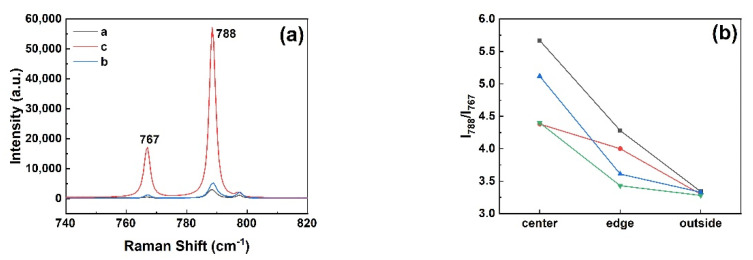
(**a**) Raman spectra of 6H-SiC micropipes; (**b**) intensity ratio of the peak at −788 cm^−1^ and the peak at −767 cm^−1^ at different positions.

**Figure 11:**
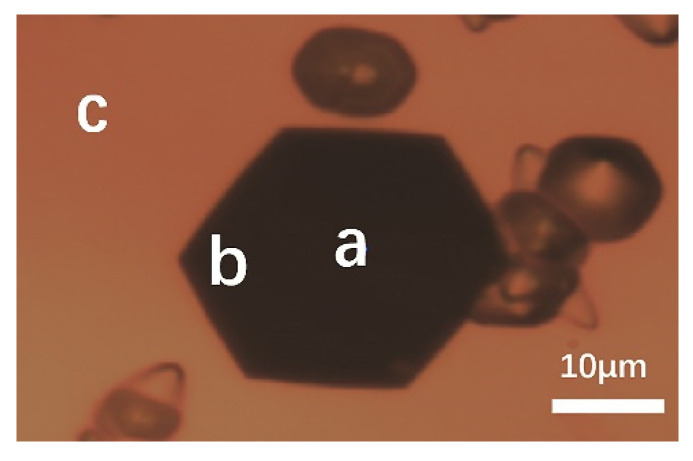
Different positions of testing: (**a**) center of a micropipe; (**b**) edge of a micropipe; (**c**) outside of a micropipe.

Raman spectroscopy was employed to characterize the different types of micropipe etch pits in 4H-SiC and 6H-SiC single crystals. It was worth noting that an accompanying peak appeared near −784 cm^−1^ in the Raman spectrum at the position of the 4H-SiC micropipe after etching in Figure 9a. In order to further verify whether this peak was a characteristic peak of micropipes, other dislocations such as TSDs and BPDs were also characterized. The −784 cm^−1^ peak did not appear in the Raman spectra of other dislocations (Figure 9b). The Raman spectrum of 6H-SiC is given in Figure 10a. Since 6H-SiC had a peak at −788 cm^−1^, the intensity ratio of the peak at −788 cm^−1^ and the peak at −767 cm^−1^ are compared at different positions in Figure 10b. Figure 11 shows the different positions of the test. I_788_/I_767_ increased as the distance from micropipes decreased. Therefore, the peak (approximately −784 cm^−1^) can be considered a sensitive peak to the micropipes, regardless of whether it appeared in 4H-SiC or 6H-SiC single crystals. This could be a means to distinguish micropipes from TSDs.

Raman scattering has the advantage that it can provide information on both the lattice structure and electronic properties of SiC [44]. The FTO mode in 4H-SiC and 6H-SiC is a forbidden band because of the backscattering geometry using the (0001) face. It can be activated by disorder in the stacking sequence [45]. In other words, FTO mode is sensitive to disorders in crystalline perfection. So, the −784 cm^−1^ peak may originate from greater disorder in the micropipe etch pit area in the process of etching.

**Figure 12:**
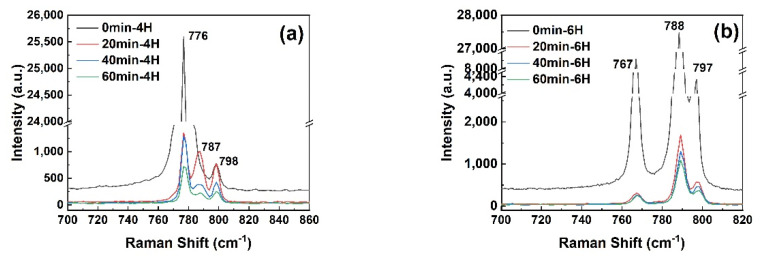
Raman spectra of micropipes etched for different amounts of time in 4H-SiC (**a**) and 6H-SiC (**b**).

**Figure 13:**
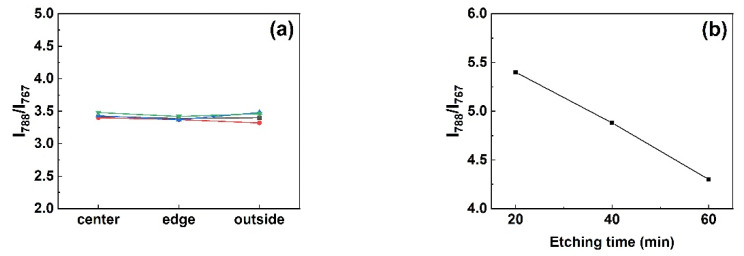
(**a**) Intensity ratio of the peak at −788 cm^−1^ and the peak at −767 cm^−1^ at different positions in unetched micropipes; (**b**) change in the intensity ratio of the peak at −788 cm^−1^ and the peak at −767 cm^−1^ with etching time.

In order to track the −784 cm^−1^ peak, Raman spectroscopy tests were also performed on the micropipes with different etching time. The result is presented in Figure 12. Figure 12a clearly shows that the −784 cm ^−1^ peak did not appear for the unetched micropipes in 4H-SiC. This was also true for 6H-SiC. According to our calculations, the value of I_788_/I_767_ did not change with the distance from the micropipe in the unetched 6H-SiC sample, as can be seen from Figure 13a. This result reveals that the −784 cm ^−1^ peak cannot correspond to unetched micropipes. This suggests that the −784 cm^−1^ peak is caused by a certain change in the micropipes during the etching process. This change may be the larger lattice disorder during the etching process mentioned above. On the other hand, the peak intensity decreased with the increase of the etching time, which might be due to the reduction of the incident laser intensity caused by the uneven surface in the inner wall of the micropipe. This was also seen in 6H-SiC (Figure 13b). The ratio of I_788_/I_767_ at the center of a micropipe decreased with the increase of the etching time, which precisely illustrates this point. In short, the −784 cm^−1^ peak is uniquely associated with the presence of micropipe etch pits.

The longitudinal optical phonon-plasmon coupled (LOPC)-mode can be used to study the electrical properties in 4H-SiC and 6H-SiC, which can fluctuate according to carrier concentration. Taking 4H-SiC as an example, a Raman shift at −967 cm^−1^ occurred in the Raman spectrum of the micropipe etch pit. The LOPC mode of the micropipe etch pits shifted to higher values. The longitudinal optical phonon and plasmon coupling mode (LOPC mode) results were analyzed to calculate the carrier concentrations of different dislocations in SiC [46]. The results are shown in Table 2. According to Table 2, we concluded that the carrier concentrations in the neighbors of the micropipe etch pits were higher than those in other dislocations and dislocation-free regions. This effect was produced by a modification of the electronic properties of the material [16].

## 4. Conclusions

We employed the wet etching method to distinguish micropipe etch pits from TSDs in 4H-SiC and 6H-SiC wafers. We confirmed that the under-etched state was the best etching condition to distinguish micropipe etch pits from TSDs. The size of the micropipe etch pits was linearly related to the etching time, and the etching rate showed a polytype dependence. The etching rate was positively related to the hexagonality in 4H-SiC and 6H-SiC. The micropipe etch pits were classified in detail. Most importantly, the spectra of 4H-SiC and 6H-SiC crystals containing micropipes were examined using Raman scattering. In the Raman spectrum of the micropipe etching pits, an accompanying peak of approximately −784 cm^−1^ was a sensitive peak of micropipe etch pits, which may be induced by the larger lattice disorder during the etching process in the area of micropipe etch pits. This work demonstrated that Raman spectroscopy was an effective way to characterize micropipes in a simple manner which might also be useful for distinguishing micropipe etch pits from TSDs.

## Figures and Tables

**Table 1:** The size difference between 4H-SiC micropipe etch pits and TSDs with etching time.

Etching Time (min)	Size of the Micropipe Etch Pits (µm)	Size of TSDs (µm)	Difference (µm)
10	15.54	11.02	4.52
20	25.62	21.84	3.78
30	35.73	32.46	3.27
40	45.81	43.14	2.67
50	56.04	53.91	2.13
60	67.04	65.63	1.41

**Table 2:** LOPC modes and carrier concentrations of different dislocations in 4H-SiC.

Defects Types	LOPC Mode (cm^−1^)	Carrier Concentration (cm^−3^)
TSD	965.6	1.85 × 10^17^
BPD	965.6	1.85 × 10^17^
TED	965.2	1.35 × 10^17^
No dislocation region	965.6	1.85 × 10^17^
MP	969.3	6.40 × 10^17^

## Data Availability

Data sharing not applicable.
